# An Integrated Approach: A Hybrid Machine Learning Model for the Classification of Unscheduled Stoppages in a Mining Crushing Line Employing Principal Component Analysis and Artificial Neural Networks

**DOI:** 10.3390/s24175804

**Published:** 2024-09-06

**Authors:** Pablo Viveros, Cristian Moya, Rodrigo Mena, Fredy Kristjanpoller, David R. Godoy

**Affiliations:** Laboratory for Reliability and Process Digitalization, Industrial Engineering Department, Universidad Técnica Federico Santa María, Valparaíso 2390123, Chile; cristian.moyag@sansano.usm.cl (C.M.); rodrigo.mena@usm.cl (R.M.); fredy.kristjanpoller@usm.cl (F.K.); david.godoy@usm.cl (D.R.G.)

**Keywords:** classification, stoppages, support vector machine, artificial neural network, principal component analysis, copper crushing, maintenance, reliability in data management

## Abstract

This article implements a hybrid Machine Learning (ML) model to classify stoppage events in a copper-crushing equipment, more specifically, a conveyor belt. The model combines Artificial Neural Networks (ANNs) and Support Vector Machines (SVMs) with Principal Component Analysis (PCA) to identify the type of stoppage event when they occur in an industrial sector that is significant for the Chilean economy. This research addresses the critical need to optimise maintenance management in the mining industry, highlighting the technological relevance and motivation for using advanced ML techniques. This study focusses on combining and implementing three ML models trained with historical data composed of information from various sensors, real and virtual, as well from maintenance reports that report operational conditions and equipment failure characteristics. The main objective of this study is to improve the efficiency when identifying the nature of a stoppage serving as a basis for the subsequent development of a reliable failure prediction system. The results indicate that this approach significantly increases information reliability, addressing the persistent challenges in data management within the maintenance area. With a classification accuracy of 96.2% and a recall of 96.3%, the model validates and automates the classification of stoppage events, significantly reducing dependency on interdepartmental interactions. This advancement eliminates the need for reliance on external databases, which have previously been prone to errors, missing critical data, or containing outdated information. By implementing this methodology, a robust and reliable foundation is established for developing a failure prediction model, fostering both efficiency and reliability in the maintenance process. The application of ML in this context produces demonstrably positive outcomes in the classification of stoppage events, underscoring its significant impact on industry operations.

## 1. Introduction

In recent years, the evolution of machinery and industrial assets has brought about changes in maintenance management. This process has undergone successive transformations over time [1], driven by more complex designs, new maintenance methods, and a changing perspective on maintenance organisation. Due to this evolution, the industrial sector focusses on finding increasingly precise ways to generate maintenance plans that align with the requirements of different industries. One of the most commonly used maintenance plans is preventive maintenance, which involves prescheduled activities to minimise unexpected damage, reduce production downtime due to failures, and consequently lower associated costs [2].

Currently, to schedule maintenance activities, Industry 4.0 fosters anomaly detection by using vast amounts of data from systems, sensors, robotics, and emerging technologies such as Artificial Intelligence (AI) and Machine Learning (ML) [3]. Deep Learning-based methods such as Complementary Adversarial network-driven Surface Defect Detection (CASDD) [4] identify various types of texture defects. Specifically, CASDD consists of an encoding–decoding segmentation module with a specially designed loss measurement and a novel complementary discriminator mechanism. In addition, [5] proposes a microelectromechanical system (MEMS) action recognition method based on Relief-F feature selection and relief-bagging-Support Vector Machine. Feature selection using the Relief-F algorithm reduces the dimensionality and the optimisation time. Experiments show that the improved algorithm for identifying non-normal walking actions presents higher accuracy compared with decision trees (DTs), k-nearest neighbours (KNN) and random forest (RF). Moreover, [6] analysed some features of students’ amounts of consumption on campus and their statistical characteristics and established a dataset based on smart campus card records to detect students’ abnormal activities.

However, extant anomaly detection methods are prone to the two issues listed below. First, most existing unsupervised anomaly detection algorithms are trained by fitting a central piece of the training data while disregarding the anomalous data. Second, these algorithms have poor time performance on large-scale datasets. 

As it can be observed, AI and ML are introduced as solutions to improve existing maintenance practices [7]. Maintenance management and reliability analysis are critical in the operation of industrial equipment, largely due to the inherent challenges of data management in this context. A fundamental problem is the lack of integration and relationship between the maintenance and operations databases. This disjunction impedes properly discriminating between operational stoppage events, failures, and unscheduled shutdowns. On the other hand, manipulation of maintenance data by human operators represents a significant challenge that leads to a decrease in the reliability of the information. 

This application may become a significant innovation for the national industry. Optimising the existing crushing process has the potential to bring about a change in mining and copper production. Considering that Chile is the world’s leading producer of this mineral [8], contributing 14.6% [9] to the country’s GDP, proper implementation of ML techniques for stoppage classification can offer significant benefits due to faster responses to operational issues, thus reducing downtime, potentially improving efficiency, and benefiting the mining industry.

The benefit of applying ML to the process is processing large volumes of data in real time. In this context, ML techniques such as Artificial Neural Networks (ANNs) [10,11,12] and Support Vector Machines (SVMs) [11,12,13,14] stand out as highly effective tools for data classification. This implementation is not only the application of advanced algorithms, but it also involves organisational change as well, where it is critical to understand that these models are constantly evolving as new data are incorporated and they adapt to the changing dynamics of assets and equipment. 

This study introduces a novel hybrid ML model that integrates ANN and SVM with PCA for the classification of maintenance events in copper-crushing equipment. Two hybrid models will be compared: PCA-ANN and PCA-SVM. The objective of this research is to enhance the accuracy and efficiency of identifying operational shutdowns and equipment failures, which are critical to optimising maintenance management in the mining industry. Unlike traditional approaches, this methodology not only automates the classification process but also significantly improves the reliability of the data by reducing dependence on manual input and external databases. The results achieved, with an accuracy rate surpassing 96%, demonstrate the potential of this approach to serve as a foundational model for developing predictive maintenance systems, ultimately driving operational efficiency and reducing costs in the mining sector.

## 2. Literature Review

To address the research problem and its development, it is necessary to understand the concept of preventive maintenance policies, along with the ML techniques and complimentary performance metrics employed throughout this research. 

### 2.1. Preventive Maintenance

Maintenance is the combination of all technical, administrative and managerial actions during the life cycle of an item intended to retain it in, or restore it to, a state in which it can perform the required function [15]. In this context, different maintenance policies, such as preventive and condition-based policies [16], define the guidelines based on which a company is to plan all its maintenance actions. This article focusses on preventive maintenance [17], which is the set of maintenance activities performed at predetermined time intervals or according to prescribed criteria to reduce the probability of failure or degradation of the functionality of an item. In addition, predictive maintenance aims to predict the optimal time point for maintenance actions, considering information about the system’s health state and historical maintenance data [18]. 

In recent years, condition monitoring systems for industrial equipment have become conventional due to lower costs and the increased reliability of sensors, data transmission, and storage devices. Simultaneously, the IoT has enabled real-time transmission of this information on system conditions captured by different monitoring devices enabling fault detection [19]. This development offers an excellent opportunity to use condition monitoring data intelligently within predictive maintenance [20]. On the other hand, ML can be interpreted as the science of using computer algorithms to perform a specific task by the use of data without applying explicit instructions [21].

### 2.2. Artificial Neural Networks (ANNs)

Artificial Neural Networks are mathematical models inspired by the biological behaviour of neurons and how they are organised to form the brain’s structure [22]. They can be explained as a simplified model of the brain; this method is used to solve classification (pattern recognition, feature extraction, and image matching) and prediction problems.

**Figure 1:**
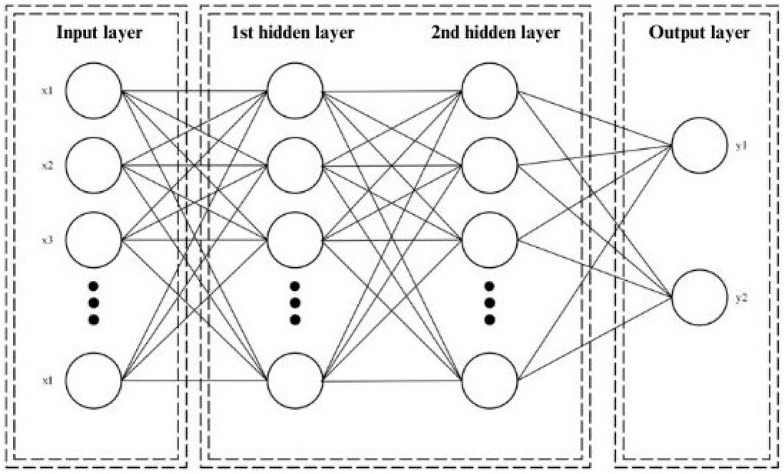
Representation of an Artificial Neural Network with two hidden layers.

The typical model is composed of layers with different perceptrons as represented in Figure 1. A perceptron, equivalent to an artificial neuron, is the fundamental unit of the network which handles computations to detect features. These layers include the input layer, which receives the input data or input features; the hidden layers, which processes the input of each perceptron from the preceding layer through a weighted sum and an appropriate activation function, which mathematically defines whether or not to send a signal to the next layer; and the output layer, which produces the network’s response [23].

The following equation mathematically represents the output of the model:

(1) $$y=f\left(x\right)=\sum_{j=1}^{J}w_{j}\delta \left(\sum_{i=1}^{I}w_{ij\;}x_{i}+b_{j}\right)+\beta +\varepsilon$$

where *x* is the l-dimensional input vector $w_{ij}$ is the weight factor connecting the input neuron *i* to the hidden neuron j, $w_{j}$ is the weight factor connecting the hidden neuron *j* to the output neuron, β is the bias for the output neuron, ε is a random error, and δ is the activation function.

### 2.3. Support Vector Machine (SVM)

**Figure 2:**
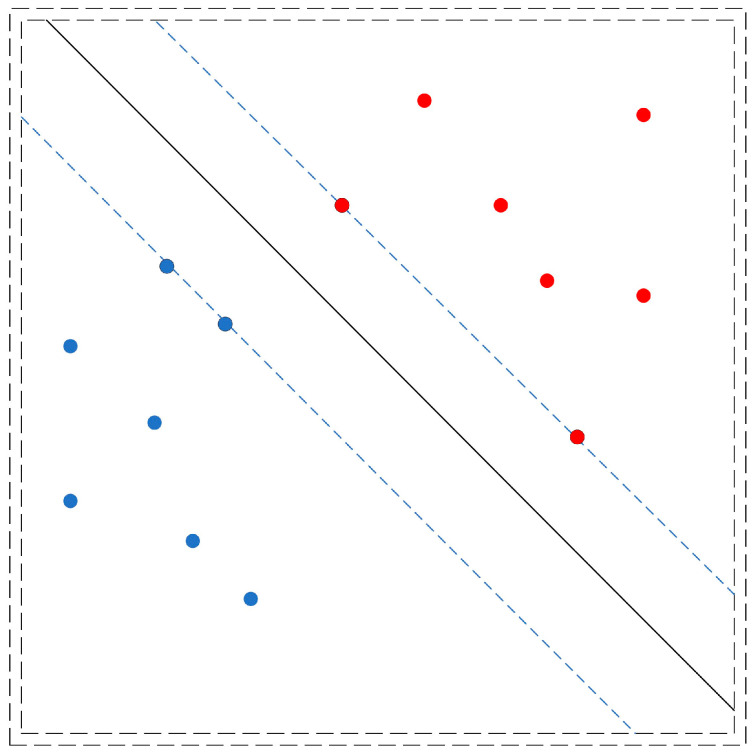
Representation of an SVM classification.

This statistical method was introduced by Vladimir Vapnik around 1960 and was initially focussed on binary classification, where hyperplanes separate data. SVM has been extended to handle multi-class classification, regression, and outlier detection. This algorithm is one of the most effective and widely used ML techniques [24]. Figure 2 shows a set of observations that fall into two classes, red and blue, SVM is able to find a hyperplane that sets a boundary between them.

This algorithm generates a separation hyperplane that maximises the margin between two datasets according to their classes. Two parallel hyperplanes are established on either side of the separation hyperplane to create the margin. The hyperplane can be described as follows:

(2) $$w^{t}x+b=0,x\in R^{d}$$

The optimal solution is obtained by reaching the highest possible margin. To find the optimal separation hyperplane, the SVM algorithm must maximise this margin while adhering to constraints by solving the following quadratic optimisation problem [25].

(3) $$\min\frac{1}{2}{\left|\left|w\right|\right|}^{2}\;\;\;s.t.,\;\;\;\;\;y_{i}\left[w\cdot x_{i}+b\right]\geq 1\;\;\;\;\;\;\;\;\forall i\;\;\;$$

Equation (3) is for data that can be separated linearly; this article considers data that are separated nonlinearly, making it necessary to perform a transformation on the data, to find a hyperplane that classifies the categories. The mathematical function used for the transformation is the kernel function k(x,y) [26]. The kernel function transforms the original input data to be converted into a higher-dimensional feature space. These kernel functions are essential to the operation of SVMs as they are able to address nonlinear classification problems, where a hyperplane in the original space cannot effectively separate the data. In general, a kernel function projects the data from a low-dimensional space to a higher-dimensional space [27]. There are different types of kernels, and in this study, three will be applied to select the one that provides the best results. The selection of the appropriate kernel function is crucial because the kernel defines the feature space in which the training set examples will be classified [28]. The selected kernel functions are as follows:

Of the various kernels available in SVM, using linear, polynomial, and radial kernels among the multiple options available in SVM is based on their broad applicability and versatility in most classification problems. Limiting to these three kernels simplifies the model selection and tuning process without sacrificing the ability to address linear and nonlinear classification problems.

Linear Kernel:

(4) $$k\left(x,x_{j}\right)=x^{t}x_{j}$$

Polynomial Kernel:

(5) $$k\left(x,x_{j}\right)={\left(\gamma x^{t}x_{j}+r\right)}^{d},\;\gamma >0$$

Gaussian Radial Basis Function (RBF) Kernel:

(6) $$k\left(x,x_{j}\right)={\exp(-\gamma \left|\left|x-x_{j}\right|\right|}^{2}),\gamma >0$$

### 2.4. Principal Component Analysis (PCA)

A fundamental tool in modern data analysis, employed across multiple disciplines, is Principal Component Analysis (PCA). Introduced by Karl Pearson in 1901, it is a linear projection that minimises the average projection cost, defined as the mean squared distance between the data points and their projections [29].

As a dimensionality reduction technique, PCA transforms a set of possibly correlated variables into a smaller set of new orthogonal variables called principal components. The values of these new variables for the observations are referred to as factor scores and can be interpreted as projections of the observations onto the principal components [30]. This technique is particularly useful for visualising and exploring high-dimensional datasets, as it can easily identify trends, patterns, or outliers [31]. PCA is effective for data preprocessing in ML algorithms, as it extracts the most informative features from large datasets while preserving the most relevant information from the initial dataset [32]. By projecting a high-dimensional dataset into a smaller feature space, PCA minimises or eliminates common issues such as multicollinearity and overfitting.

The mathematical formulation [30] begins with the following: Let $X=[x_{1},\ldots ,x_{n}]$ be the dataset to be analysed using Principal Component Analysis (PCA), where each column represents a single observation described by M variables. The sample mean vector $\bar{x}$ and the sample covariance matrix ∑ can be represented as follows:

(7) $$\bar{x}=\frac{1}{N}\sum_{i=1}^{N}x_{i}$$

(8) $$\sum =\frac{1}{N}\;\;\sum_{i=1}^{N}(x_{i}-\bar{x}\;){\left(x_{i}-\bar{x}\right)}^{T}=\frac{1}{N}\;\tilde{X\;}{\overset{ˇ}{X}}^{T}$$

where $\tilde{X}=\left[x_{1}-\bar{x},\;\ldots ,\;x_{N}-\bar{x}\right].$ PCA computes factor scores as linear combinations of the original variables.

(9) $$y_{1i}=a_{1}^{T}\left(x_{i}-\bar{x}\right),\;\;\;\;\left(i=1,\ldots ,N\right)\;\;$$

The optimal weight $a_{1}$ is obtained by maximising the variance in $y_{1i}$ under the constraint $a_{1}^{T}a_{1}=1$. This leads one to find the eigenvector a1a_1a1 corresponding to the largest eigenvalue $\lambda_{1}\;of\;\Sigma :$

(10) $$\Sigma a_{1}=\lambda_{1}a_{1}$$

Additional principal components are similarly obtained as eigenvectors corresponding to subsequent largest eigenvalues. For an L-dimensional projection, the optimal projection matrix A consists of the L eigenvectors of ∑ associated with the L largest eigenvalues $\lambda_{1},\;\ldots .\;,\;\lambda_{L}:$

(11) $$Y=A^{T}\tilde{X\;}$$

(12) $$\Sigma A=A\Lambda ,\;\;\;\;\;\;\;\;\;\;\;\;\Lambda =diag\left(\lambda_{1},\;\ldots ,\;\lambda_{L}\right)$$

### 2.5. Performance Metrics

To evaluate the model’s effectiveness in classifying stoppages, metrics for comparing the different techniques and functions used are defined. One of the selected tools for evaluation includes the confusion matrix [33], which shows the number of correct and incorrect classifications made by the model on a dataset. See Table 1.

Other metrics are derived from the confusion matrix as follows [33].

Accuracy calculates the proportion of instances classified correctly (both true positives and true negatives) out of the total classifications [34]. Accuracy is a vital ML metric that quantifies the model’s overall correctness in making predictions. One of its significant advantages is its simplicity and ease of interpretation. It provides a clear, straightforward percentage of how many predictions were correct, making it a valuable metric for quickly assessing the general performance of a model.

(13) $$Accuracy=\frac{TP+TN}{TP+TN+FP+FN}\;\;\;\;$$

Precision (P) will also be used, representing the number of true positives among the total number of positive predictions, which is crucial for minimising false positives. High precision indicates a low rate of false positives, which is important in applications where false positives are costly or undesirable.

(14) $$Precision=\frac{TP}{TP+FP}$$

Recall (r) is a metric used to measure the fraction of true positive values compared to the total number of positive cases. High recall implies a low rate of false negatives, making it important when missing a positive instance has significant consequences.

(15) $$Recall=\frac{TP}{TP+FN}$$

Specificity, also known as the true-negative rate, is a fundamental metric in classification models, reflecting the model’s ability to accurately identify negative cases. It is calculated as the proportion of true negatives (TN) to the sum of the true negatives and false positives (FP). Mathematically, the formula for specificity is

(16) $$Specificity=\frac{TN}{TN+FP}$$

The F-measure (FM) can be derived from the recall and precision metrics, representing the harmonic mean between precision and recall, and provides a balanced assessment of a model’s performance, especially when precision and recall need to be balanced. It is beneficial when dealing with imbalanced datasets, as it combines false positives and negatives into a single score, helping in model evaluation and comparison.

(17) $$F-Measure=\frac{2\cdot Precision\cdot Recall}{Precision+Recall}\;$$

Performance metrics play a key role in various ML applications. These metrics are used to effectively evaluate and compare models against each other, providing a solid basis for making decisions about which model and algorithm perform best in the area of interest. Their widespread use is due to their proven effectiveness in model evaluation [35,36,37,38], which contributes significantly to informed decision making in ML. 

The choice of evaluation metrics in an ML model is paramount as it determines how performance is measured. Each metric, such as accuracy, precision, recall, and F-measure, provides a unique perspective on the model’s quality and allows for tailored evaluation based on the specific problem; thus, by combining these metrics, a balanced approach is achieved, considering the model’s accuracy and the minimisation of false positives or negatives, resulting in a more robust and effective model.

## 3. Problem Statement or Problem Formulation

**Figure 3:**
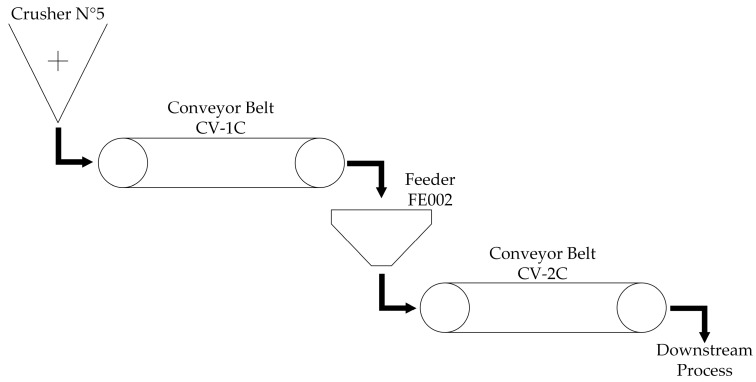
Mining crushing line.

The equipment to be investigated is a conveyor belt (CV-2C), which is part of a mining crushing line (Figure 3) that consists of a crusher where large pieces of material are reduced to smaller pieces which are later transported by conveyor belt “CV-1C” into feeder “FE002” which deposits the material into conveyor belt “CV-2C” that moves the load towards the rest of the process.

The conveyor belt CV-2C is a critical component in the crushing and material conveying line. Its correct operation is essential for maintaining the efficiency and productivity of the process. However, the conveyor belt has experienced several stoppages due to failures or operational decisions which are not informed in a timely manner. This has led to several delays when attempting to resume operation due to the unknown nature of the stoppage, which gives rise to the need for research on a classification model that can be later integrated into failure prediction systems, providing valuable input to anticipate problems, improve maintenance management, and reduce unplanned downtime.

The main identified failure modes of the CV-2C conveyor belt include the following:

- Electrical failure: Problem related to the belt’s electrical system;
- Mechanical failure: Wear or damage to mechanical components;
- Pulley misalignment: Misalignment of pulleys;
- Belt cut: Physical damage to the belt;
- Belt misalignment: Misalignments in belt alignment;
- Damaged idler: Damage to the idlers supporting the belt;
- Alignment rod failure: Problems with the belt alignment rod.

To address the conveyor belt problem, several critical variables that affect its operation are identified and monitored. Monitored data are collected through a combination of physical and virtual sensors:

Physical sensors: Embedded into the system and monitored by a Distributed Control System (DCS), they directly measure operational variables such as motor load, speed, and power. These sensors are certified and regularly maintained by a third-party company.

Virtual sensors: Generated by Simulation Digital Twin software, these sensors provide belt stress information and operational status which are estimated from the DCS data through internal algorithms.

Key parameters obtained from the physical and virtual sensors include the following:

- Belt load (tph): Measured in tons per hour, it indicates the amount of material conveyed;
- Belt speed (%): Percentage of belt operating speed;
- Belt operating status: Binary indicator showing whether the belt is running or has stopped;
- Motor power (kW): Power consumed by the motors driving the belt;
- Belt strength (kN): Measurement of the actual and design strength of the belt.

## 4. Methodology

**Figure 4:**
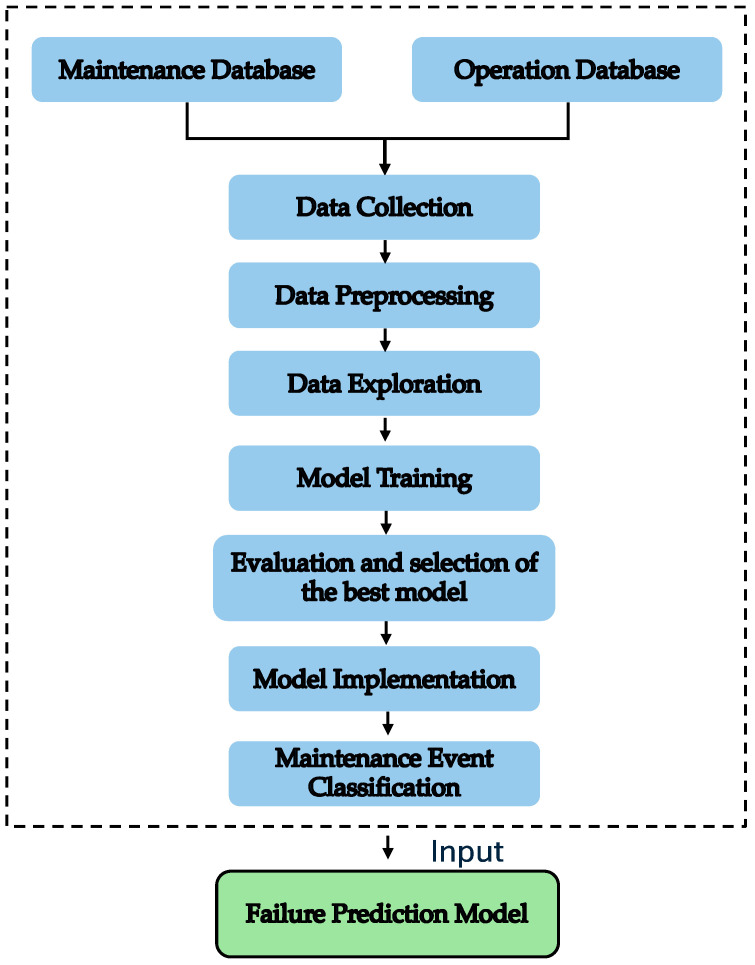
General methodology.

This section will detail the step-by-step process, including data collection and analysis, along with the key aspects required to achieve the desired results. Figure 4 depicts the overall process, where the elements in blue represent the processes and stages carried out in this article. The process starts with data collection from the DCS and Simulation Digital Twin software, followed by a rigorous data preprocessing and exploration development and optimisation. Once the data are prepared, two hybrid models are trained and compared to select the best classification model.

The transition from the classification models, shown in blue, to the “Failure Prediction Model”, highlighted in green, underlines the continuity of this research from the current approach towards a future application in failure prediction.

### 4.1. Data Collection

**Figure 5:**
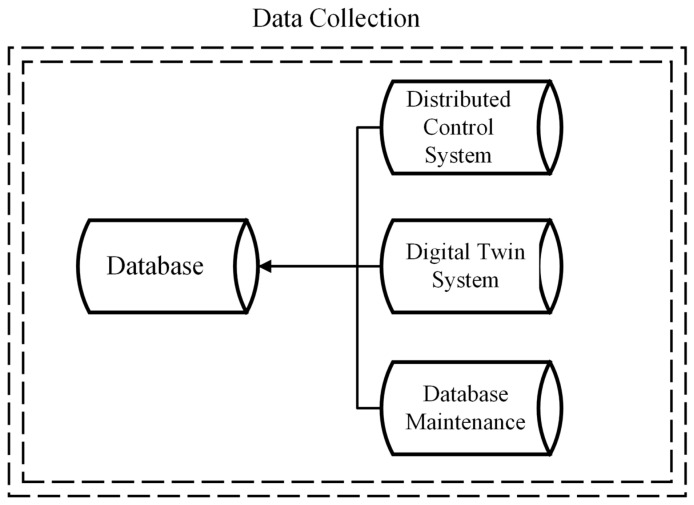
Different sources of this study’s database.

The article’s general methodology commences with data collection. The database comprises data related to the history of equipment stoppages, as well as information concerning voltage variables and the operational status of the component. This information is obtained from three sources, as represented in Figure 5: the Distributed Control System (DCS), a control infrastructure employed in industrial settings to oversee and manage various operational parameters [39]; the Digital Twin (DT) system, a virtual emulation of real-world systems or processes, reproducing variables and conditions [40]; and the maintenance database, which houses historical stoppage information.

The DCS informs the measures of the parameters shown in Table 2 while the DT informs accurate estimations of the parameters represented in Table 3, and the maintenance database reports the maintenance stoppages information described in Table 4.

To ensure the quality of data collected from the maintenance and operations databases, quality control measures were implemented. These included data integrity verification procedures, such as duplicate checking and cross-consistency reviews with technical experts to ensure data accuracy and relevance. These practices sustain the credibility of the analysis and its findings.

By cross-referencing the databases, an extra Boolean label was included on the unified database that classifies all the stoppage events recorded during the period analysed in two main categories: operational stoppage and failure.

As shown in Table 5, eleven different classes of stoppages have been identified, of which three correspond to operational stoppages and eight are identified as equipment failure.

### 4.2. Data Preprocessing

**Figure 6:**
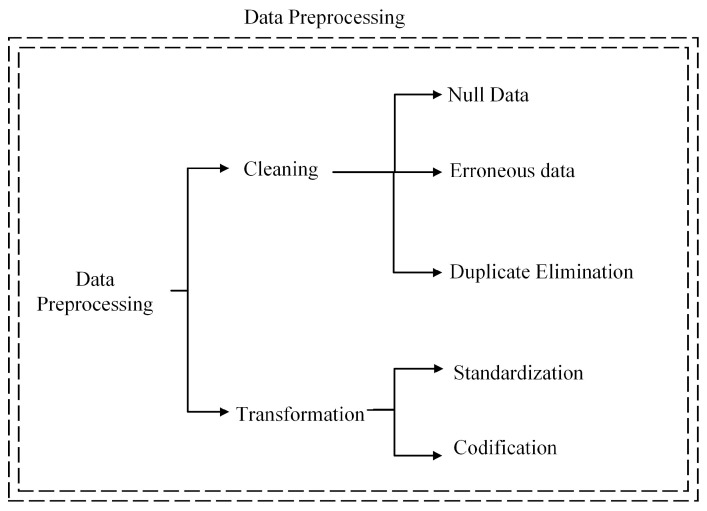
Database preprocessing.

After completing data collection, the next step in the methodology is data preprocessing. This article mainly divides this process into two stages: data cleansing and transformation. To better understand the key actions to be carried out in these stages, they can be visualised in detail in Figure 6.

The database contains 511,265 historical data points, from which only 41,447 records correspond to stoppages of the CV-2C conveyor belt and will be considered for this analysis. In the first part of the preprocessing, data cleaning was performed on the database; this cleaning considered the removal of null or empty data, erroneous data, and duplicates from the database. As a result of this cleaning, the number of data points was reduced from 41,447 to 40,596 valid records.

With the selected data, the second part of the preprocessing is performed, where the search for outliers, which are defined as points lying three standard deviations away from the mean, was conducted. Each detected outlier is carefully examined in the context of domain-specific knowledge. This makes it possible to distinguish between measurement errors and legitimate operational variations and decide on the appropriate action for each case, either eliminating or correcting these values, thus minimising the risk of bias in subsequent analyses.

During the transformation phase, the data are normalised to improve the accuracy of the models and avoid scaling problems between the numerical entries of the database. In addition, Boolean entries were numerically coded for the use of the ML algorithms.

### 4.3. Data Exploration

An exploratory data analysis was performed on the database to understand the characteristics and possible patterns in the data. Key variables, such as component operating conditions and stress indicators, were examined to identify trends, correlations, and any relevant information that could contribute to the accurate classification of events.

Measures of central tendency, such as mean, variance, and median, were calculated to examine the behaviour of each of the variables. These measures provide important information about the data’s characteristics. However, these statistics alone may not reveal complex relationships and patterns within the dataset, especially when dealing with multiple variables. Table 6 summarises the key statistical characteristics of the most relevant entries from the database.

From these initial analyses, no clear conclusion could be drawn, which could be explained by relationships between the variables that are not readily apparent through traditional summary statistics and visualisations.

In addition, a correlation analysis revealed that most variables share some significant correlation, which is a problem when employing traditional methods such as linear regressions due to the negative effect that multicollinearity has on the variance. 

However, correlation analysis assistance has limitations, as it focusses on pairwise relationships and may not capture higher-order dependencies or collinearities between multiple variables.

### 4.4. Training Model

Due to the high correlation between variables and the need to capture dependencies, PCA is employed, enabling dimensionality reduction in the dataset while preserving the most critical information. It computes and proposes a set of uncorrelated linear combinations of the original observations of the dataset to describe most of the variability of the data.

The next step is to split data for model training. This is performed randomly, splitting, 80% for training and 20% for testing. The division of the data into training and test sets was performed randomly to preserve the independence and representativeness of the samples. 

An ANN was implemented composed of an input layer, two hidden layers and an output layer. The first hidden layer contains 64 perceptrons with a ReLU (Rectified Linear Unit) activation function, while the second hidden layer has 32 perceptrons also with ReLU activation. The output layer uses a sigmoid activation function for binary classification of the maintenance events. The model was trained for 1000 epochs.

For comparison with the ANN, an SVM algorithm was implemented. In this regard, 3 different kernels were tested with 4 different C-values each. All the steps of the methodology were implemented using Python 3.10.9.

## 5. Results and Discussion

This section presents results from the experimental data to evaluate the performance of the stoppage classification models. Individual results will be presented for each of the two algorithms, and a comparison will be provided.

Classifying between shutdowns caused by unexpected equipment failures and scheduled shutdowns is crucial for several reasons and enables organisations to optimise maintenance and operation strategies. Unplanned outages are often costly and disruptive, and the ability to predict them can prevent economic losses and ensure uninterrupted production.

The configuration of the equipment used to carry out this study is essential for understanding the context in which this research was developed and could be improved with greater capabilities. The setup for the computations of this investigation was a desktop computer running Python 3.10 with Windows 10 64-bits on an Intel Core i5-7200 processor, accompanied by a RAM of 16 GB.

### 5.1. Principal Component Analysis

Before presenting the results obtained with SVM and ANN, it is important to highlight that in this study Principal Component Analysis (PCA) was implemented as a dimensionality reduction technique in conjunction with these algorithms, the combination of PCA with ML can improve the accuracy and efficiency of the models [41], in addition to avoiding multicollinearity problems [42]. PCA was applied to mitigate data complexity by removing redundant or irrelevant features, thus allowing a more efficient representation of critical features for failure event classification.

**Figure 7:**
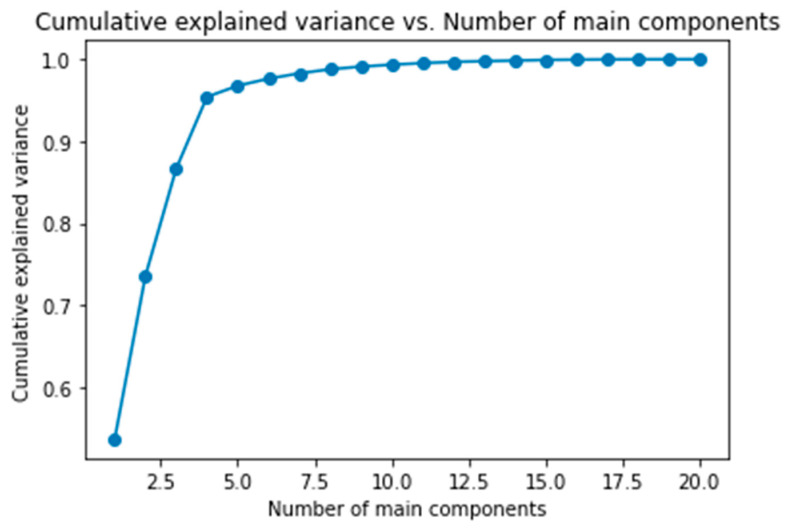
Number of main components.

PCA was performed with four principal components explaining over 90% of the variance (see Figure 7). In addition to improving model accuracy, PCA helped significantly reduce processing times, particularly in industrial applications where operational efficiency and real-time decision making are imperative; when applying PCA, the results achieved using SVM and ANN are presented in Section 5.2 and Section 5.3.

**Figure 8:**
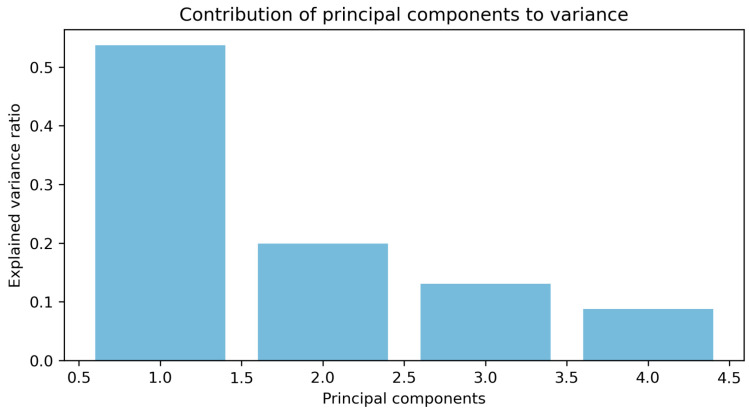
Contribution of principal components to variance.

The graph in Figure 8 clearly shows the percentage of variance explained by each of the four principal components selected.

The first four coefficients with their corresponding wights of the four selected principal components are presented in Equations (18)–(21) as follows:

(18) $$\begin{array}{c} PC1=0.2608\times Total\;Power\left(DT\right)\\ \;+0.258\times Utilization\;Percentage\;of\;Power\;Capacity\left(DT\right)\\ \;+0.257\times Torque\;\left(DT\right)+0.2545*Current\;of\;Motor\;1\left(DT\right)+\cdots \end{array}$$

(19) $$\begin{array}{c} PC2=0.200\times Belt\;Load\left(DT\right)+0.2009\times Total\;Power\left(DT\right)\\ \;+0.1988\times Utilization\;Percentage\;of\;Power\;Capacity\left(DT\right)\\ \;+0.1966\times Current\;of\;Motor\;1\left(DT\right)+\cdots \end{array}$$

(20) $$\begin{array}{c} PC3=0.4101\times Power\;of\;Motor\;1\;\left(DCS\right)\\ \;+0.4026\times Power\;Factor\;of\;Motor\;2\;\left(DCS\right)\\ \;-0.3987\times Power\;Factor\;of\;Motor\;1\;\left(DCS\right)\\ \;+0.3842\times Current\;of\;Motor\;2+\cdots \end{array}$$

(21) $$\begin{array}{c} PC4=-0.5402\times Current\;of\;Motor\;2\;Error\;\left(DT\right)\\ \;-0.5127\times Current\;of\;Motor\;1\;Error\;\left(DT\right)\\ \;+0.0918\times Belt\;Load\;Error\;\left(DT\right)\\ \;-0.0846\times Current\;of\;Motor\;2+\cdots \end{array}$$

To better understand the complexity of the operational data and how certain factors contribute to the overall dynamics of the system, a principal component composition analysis was conducted to elucidate the impact of operational factors on system dynamics. This analysis revealed that PCA tends to prioritise variables related to the level of use of the belt, such as the employed power, current, and produced torque, which is expected due to the nature of the process and the system under study.

### 5.2. Support Vector Machine

For this algorithm, an analysis of the values obtained for different kernels and various values of C was performed, where C controls the penalty for classification errors. The results obtained for different combinations are presented in Table 7.

**Figure 9:**
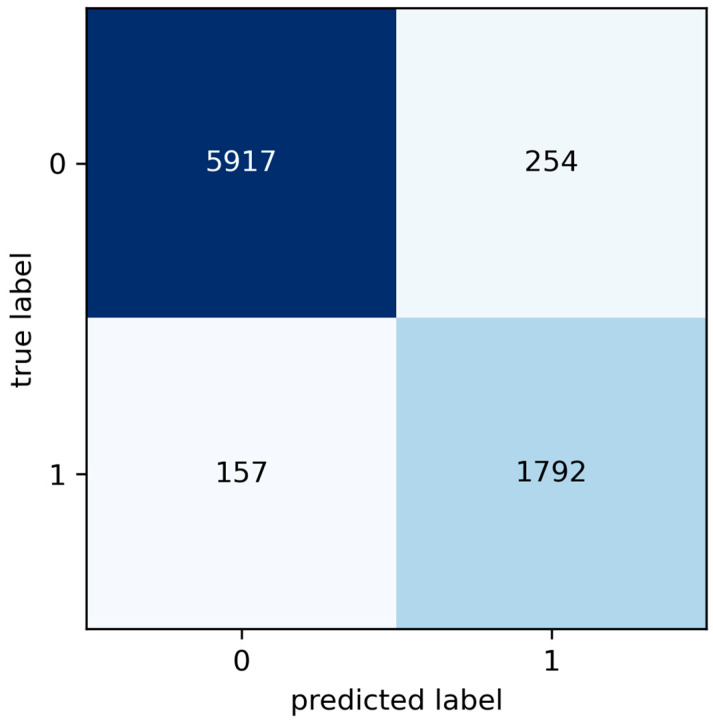
Confusion matrix for SVM classification with RBF kernel and C = 100.

In Table 7, the selected kernel for developing the model is RBF with a C of 100, which yields an accuracy of 94.94%. The confusion matrix of this setup is shown in Figure 9.

The confusion matrix in Figure 9 reveals critical aspects of the model’s performance in classifying stoppage events. Among the analysed events, 5917 were correctly identified as operational stoppages and 1792 as failures, demonstrating the model’s ability to discern between the two types of stoppages. However, significant deviations were observed: 254 operational stoppages were erroneously classified as failures, constituting type I errors or false positives. This type of error can lead to unnecessary maintenance actions, increasing operational costs. Additionally, 157 actual failures were not detected and were incorrectly classified as operational stoppages, representing type II errors or false negatives, which could result in a lack of necessary maintenance and potentially critical equipment failures. The precise distinction between these types of errors is fundamental for the continuous optimisation of the maintenance process and the improvement in the model’s reliability. The other metrics that will be used to evaluate and compare the model are presented in Table 8.

Table 8 confirms that even though SVM is capable of high accuracy, it does not excel in specificity, which may lead to a costly number of false positives.

### 5.3. Artificial Neural Network

**Figure 10:**
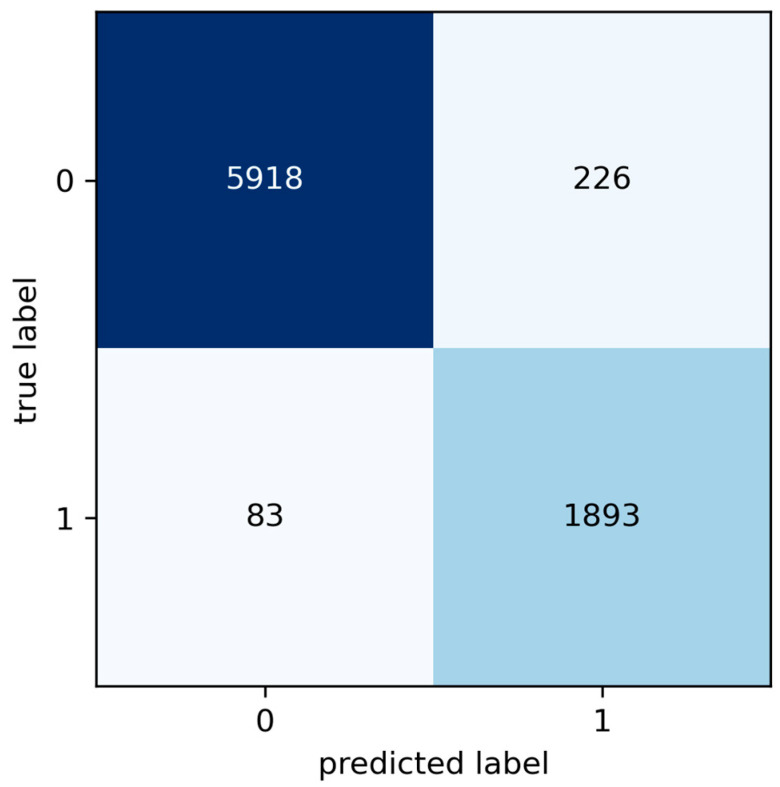
Confusion matrix of the ANN.

After training under the same setup, the performance of the Artificial Neural Network (ANN) in classifying stoppage events is analysed. Figure 10 shows the confusion matrix regarding the performance achieved by the ANN in this task. The confusion matrix breaks down the classification performed by the model, where 5918 events were correctly identified as operational stoppages and 1893 as failures, demonstrating the algorithm’s effectiveness in distinguishing between these critical categories. However, there were some significant misclassifications: 226 operational stoppages were incorrectly labelled as failures, representing Type I errors. This error suggests an over-sensitivity of the model towards detecting failures, which can lead to unnecessary and costly maintenance interventions. Conversely, 83 actual failures were incorrectly classified as operational stoppages, constituting Type II errors, posing risks to safety and reliability by not performing critical maintenance as needed.

These results highlight the importance of refining the model’s accuracy to minimise both types of errors, thereby ensuring the effectiveness and efficiency of the maintenance process. The results obtained for the performance metrics regarding the ANN are presented in Table 9. In general, performance is improved with respect to SVM. The ANN was able to improve the scores obtained in precision and specificity, hinting that false positives were less common when using an ANN.

### 5.4. Processing Time

Another aspect of relevance in the analysis of the results is the processing time required by each of the classification algorithms when PCS is conducted. This factor, fundamental in evaluating the proposed solutions’ efficiency, is presented in detail in the table showing the execution time before and after applying the PCA for the ANN and SVM algorithms. Reducing processing time, one of the key advantages of PCA, becomes a prominent element in evaluating the feasibility of implementing these models in an industrial environment.

The processing time results are positive, as a drastic decrease in the time required by both algorithms is observed after applying the PCA. Compared to previous runtimes, a substantial reduction of more than 40% in the time required to complete the classification tasks has been achieved. The optimisation of the processing time, due to PCA, is presented in Table 10.

### 5.5. Comparison 

Confusion matrices for the ANN and SVM models provide essential insights into the effectiveness of these models in classifying maintenance events and the types of common errors that arise during classification. Classification errors manifest as false positives (FP) and false negatives (FN). An FP occurs when the model incorrectly predicts a failure in an operational shutdown instance; conversely, an FN occurs when an actual failure is misclassified as an operational shutdown. These errors are critical as they can lead to unnecessary maintenance or operational shutdown instances needing maintenance, thereby increasing costs and unplanned downtime.

Specifically, the SVM model recorded 254 false positives (Type I errors) and 157 false negatives (Type II errors), while the ANN model showed 226 false positives and 83 false negatives. These differences suggest that, although both models are effective, the ANN has a slightly better rate of reducing Type II errors, which is crucial for avoiding skipped maintenance. This reduction in false negatives is essential for ensuring that all necessary maintenance tasks are performed, and no critical failures are overlooked, thus maintaining operational integrity and safety. 

Regarding the performance metrics, Table 11 presents the results from both classification algorithms applied. It is observed that, overall, the ANN performed better than the SVM. In accuracy, the ANN shows more favourable results where 96.2% of the analysed stoppages are correctly categorised. For SVM, the specificity is 91.9%, and for ANN, it is four points higher at 95.8% indicating that ANN is more effective in accurately rejecting non-failure events, minimising the unnecessary interventions and maintenance that could result from incorrectly flagged operational stoppages. This capability is crucial in industrial settings where false alarms can lead to costly and disruptive maintenance actions that are not needed. The precision of the ANN scored 98.6% in the correct categorisation of failures while the SVM scored 97.4%, showing that ANN is also better suited for reducing false positives. For recall, the ANN scored 96.3%, indicating that the ANN is more effective in capturing most of the real fault events, minimising false negatives. For the F1-score metric, neural networks also show better results with 97.4%, which implies a good balance between accuracy and recall, indicating fewer errors in fault classification and stoppages.

Another critical aspect to be evaluated to achieve this article’s objective is the selected model’s processing time. In this regard, the ANN has shown more favourable performance than SVM as it outperforms SVM in every score from the selected metrics, while at the same time achieving lower computational times. This is not even considering that ANNs are also optimised to run on graphics cards which may reduce the processing time of ANNs into a fraction of the original time. 

Since this article aims to facilitate real-time implementation of the classification model, ensuring time efficiency is crucial. This efficiency allows for swift decision making, which is essential for maintaining operational continuity in industrial settings.

The ANN can learn complex patterns and relationships in the data, enabling accurate and dynamic classification. Combining PCA with ANN facilitates data dimensionality reduction, improving model efficiency and reducing processing times without sacrificing precision and accuracy. Predicting unplanned outages is critical to asset management and operational efficiency. This allows informed decisions to be made in real time and paves the way for developing more advanced failure prediction models. These models can effectively classify maintenance events within the copper-crushing line equipment context. Moreover, ANN’s can successfully classify stoppages in a very short time, which may be the first step into the development of a real-time prediction tool.

In the context of this case study, the results highlight the superior performance of the ANN, which consistently outperformed the other algorithms in all the evaluated metrics. With accuracy, precision, recall, and F1-score values consistently exceeding 95%, ANNs demonstrate their remarkable ability to identify and categorise stoppage events while minimising misclassifications accurately. This exceptional performance underscores their potential as a valuable tool for similar tasks within the industry.

## 6. Conclusions

This article presents and evaluates the application of two ML algorithms—Artificial Neural Networks (ANNs) and Support Vector Machines (SVMs)—together with Principal Component Analysis (PCA) to create a hybrid model. Both the tested approaches effectively classify the nature of the stoppages within the copper-crushing line equipment context, ultimately serving as a foundational component for future failure prediction models. This forward-looking approach holds significant potential for maintenance practices within the mining industry and across various sectors that rely on sensor data and stress-related variables for equipment.

The importance of this application in the mining industry is highlighted by its ability to improve the efficiency of existing processes, which have historically been plagued with maintenance management problems due to manual data collection, resulting in error-prone data. By focussing on classification, based solely on data generated by operational sensors, this research provides more reliable information for prediction. As such, it lays the foundation for creating a more reliable failure prediction model, thereby reducing dependence on external sources and mitigating potential problems such as inaccuracies, data gaps, and outdated information. Ultimately, this methodology has the potential to significantly improve operational efficiency and reduce costs in the mining industry, consolidating its relevance and positive impact on the sector.

In the context of this case study, the results highlight the superior performance of the ANN, which consistently outperformed the other algorithms in all evaluated metrics. With accuracy, precision, recall, specificity, and F1-score values consistently exceeding 95%, ANNs demonstrate their remarkable ability to identify and categorise stoppage events while minimising misclassifications accurately. The specificity achieved in the models, particularly the ANN’s superior ability to correctly reject non-failure events as non-failures, demonstrates the model’s effectiveness in avoiding unnecessary maintenance actions and underscores the reliability of the classification system in operational environments.

Another critical aspect examined in this article is the processing time of the selected model where the ANN proved to be more time-efficient than the SVM, a significant advantage given the real-time implementation required for effective classification. Furthermore, as mentioned, current implementations of ANN can leverage the computational power of graphic cards (GPU) to reduce the computational times by fractions of the processing time in a conventional CPU.

In light of the encouraging results of this research, it is strongly recommended that this methodology and model are implemented within copper-crushing line equipment and their extension to other equipment is explored. This methodology effectively leverages the benefits of ML, enabling the processing of substantial data volumes in real time and enhancing early event classification, thus contributing to significant process efficiency improvements.

In conclusion, this study has successfully developed a hybrid ML model capable of identifying and categorising unscheduled stoppage events in the equipment database of a Chilean mining company. This achievement not only fulfils the primary research objectives but also lays the foundation for the future integration of this methodology into advanced failure prediction models. As a result, it offers the potential to enhance the reliability of preventive maintenance practices, ultimately improving the operational efficiency and cost effectiveness of industrial processes.

## Figures and Tables

**Table 1:** Confusion matrix.

	Actual Positive Class	Actual Negative Class
Predicted positive class	True positive (TP)	False negative (FN)
Predicted negative class	False positive (FP)	True negative (TN)

**Table 2:** Parameters reported by the DCS.

Parameter	Type	Units
working status	boolean	
belt load	numerical	TPH
current speed percentage of top speed	numerical	
current of motor 1	numerical	amp
current of motor 2	numerical	amp
power factor of motor 1	numerical	
power factor of motor 2	numerical	
power of motor 1	numerical	kW
power of motor 1	numerical	kW

**Table 3:** Estimations reported by the DT.

Parameter	Type	Units
belt load	numerical	TPH
belt load error	numerical	TPH
current of motor 1	numerical	amp
current of motor 2	numerical	amp
current of motor 1 error	numerical	amp
current of motor 2 error	numerical	amp
total power	numerical	kW
utilisation percentage of power capacity	numerical	
utilisation percentage of voltage capacity	numerical	
current speed percentage of top speed	numerical	
torque	numerical	kNm

**Table 4:** Maintenance database stoppages description entries.

Parameter	Type	Units
stoppage start time	numerical	
stoppage finish time	numerical	
stoppage duration	numerical	hours
unavailable time	numerical	hours
equipment id	text	
stoppage reason	text	
shift (day/night)	text	
stoppage code	text	
production line id	text	

**Table 5:** Stoppage classification.

Event	Category
Operational stoppage	Scheduled stoppage
	Downstream unscheduled stoppage
	Unscheduled upstream stoppage
Failure	Unscheduled stoppage
	Damaged alignment staff
	Electric belt cutting
	Pulley misalignment
	Failure pull chord
	Belt misalignment
	Damaged idler
	Unidentified mode

**Table 6:** Exploratory data analysis summary.

Description	Count	Mean	Std. Dev.	Min	25%	50%	75%	Max
Belt load in tph from DCS	40,596	8.5	418.2	0	1	0.3	0.4	9499
Belt load in tph from DT	40,596	615.1	2197.2	0	55.1	200	650	994.5
Power DT (two motors)	40,596	195.5	677.3	0	35.2	120	300	5791.1
Current deviation for motor 2	40,596	−2.3	5.9	−6.1	−0.8	0.3	0.8	28.9
Belt speed in % from DT	40,596	10.4	29.5	0	0	0	0	100
Torque DT	40,596	2.1	7.2	0	0	0	0.3	60
Actual belt resistance	40,596	2.2	7.4	0	0	0	0.8	61.3
Performance tonnes per shift	40,596	250	75	100	185	250	315	400
Percentage of conveyor belt Speed DCS	40,596	75	15	50	65	75	85	100

**Table 7:** SVM accuracy for different kernels and values of C.

Accuracy				
Kernel	C = 10	C = 50	C = 100	C = 250
Linear	79.48%	78.07%	56.49%	41.54%
RBF	89.16%	91.27%	94.94%	82.60%
Poly	75.13%	74.33%	53.74%	55.11%

**Table 8:** Performance metrics for the selected SVM model.

Metric	Result
Accuracy	94.9%
Precision	97.4%
Recall	95.9%
Specificity	91.9%
F1-score	96.6%

**Table 9:** ANN performance metrics.

Metric	Result
Accuracy	96.2%
Precision	98.6%
Recall	96.3%
Specificity	95.8%
F1-score	97.4%

**Table 10:** Classification processing times, in seconds, with and without conducting PCA first.

	SVM	ANN
Without PCA	89.40	83.97
With PCA	53.31	45.19

**Table 11:** Performance metric comparison for SVM and ANN.

Metrics	SVM	ANN
Accuracy	94.9%	96.2%
Precision	97.4%	98.6%
Recall	95.9%	96.3%
Specificity	91.9%	95.8%
F1-score	96.6%	97.4%

## Data Availability

The data presented in this study are available on request from the corresponding author.

## Acronyms

AI	Artificial Intelligence
ANN	Artificial Neural Network
DCS	Distributed Control System
DT	Digital Twin
FM	F-measure
FN	False negative
FP	False positive
IoT	Internet of Things
ML	Machine Learning
PCA	Principal Component Analysis
RBF	Radial Basis Function
SVM	Support Vector Machine
TP	True positive
TN	True negative

## Nomenclature

x	l-dimensional input vector
$w_{ij}$	Weight factor connecting the input neuron *i* with the hidden neuron *j*
$w_{j}$	Weight factor connecting the hidden neuron *j* to the output neuron
β	Bias for the output neuron
ε	Random error
δ	Activation function
γ	Constant
d	Polynomial degree
$X_{n}$	Input data
P	Precision
r	Recall
k	Kernel function
C	Non-negative penalty parameter
